# French invasive Asian tiger mosquito populations harbor reduced bacterial microbiota and genetic diversity compared to Vietnamese autochthonous relatives

**DOI:** 10.3389/fmicb.2015.00970

**Published:** 2015-09-22

**Authors:** G. Minard, F. H. Tran, Van Tran Van, C. Goubert, C. Bellet, G. Lambert, Khanh Ly Huynh Kim, Trang Huynh Thi Thuy, P. Mavingui, C. Valiente Moro

**Affiliations:** ^1^Ecologie Microbienne, UMR Centre National de la Recherche Scientifique 5557, USC INRA 1364, VetAgro Sup, FR41 BioEnvironment and Health, Université Claude Bernard Lyon 1Villeurbanne, France; ^2^Laboratoire de Biométrie et Biologie Evolutive, UMR 5558, CNRS, INRIA, VetAgro SupVilleurbanne, France; ^3^Entente Interdépartementale Rhône-Alpes pour la DémousticationChindrieux, France; ^4^Entente Interdépartementale de Démoustication du Littoral MéditerranéenMontpellier, France; ^5^Department of Medical Entomology and Zoonotics, Pasteur Institute in Ho Chi Minh CityVietnam; ^6^Université de La Réunion, UMR PIMIT, INSERM U1187, CNRS 9192, IRD 249, Plateforme Technologique CYROISaint-Denis, France

**Keywords:** *Aedes albopictus*, *Dysgonomonas*, holobiont, microbiota, microsatellite, phylogeography, *Wolbachia*

## Abstract

The Asian tiger mosquito *Aedes albopictus* is one of the most significant pathogen vectors of the twenty-first century. Originating from Asia, it has invaded a wide range of eco-climatic regions worldwide. The insect-associated microbiota is now recognized to play a significant role in host biology. While genetic diversity bottlenecks are known to result from biological invasions, the resulting shifts in host-associated microbiota diversity has not been thoroughly investigated. To address this subject, we compared four autochthonous *Ae. albopictus* populations in Vietnam, the native area of *Ae. albopictus*, and three populations recently introduced to Metropolitan France, with the aim of documenting whether these populations display differences in host genotype and bacterial microbiota. Population-level genetic diversity (microsatellite markers and COI haplotype) and bacterial diversity (16S rDNA metabarcoding) were compared between field-caught mosquitoes. Bacterial microbiota from the whole insect bodies were largely dominated by *Wolbachia pipientis*. Targeted analysis of the gut microbiota revealed a greater bacterial diversity in which a fraction was common between French and Vietnamese populations. The genus *Dysgonomonas* was the most prevalent and abundant across all studied populations. Overall genetic diversities of both hosts and bacterial microbiota were significantly reduced in recently established populations of France compared to the autochthonous populations of Vietnam. These results open up many important avenues of investigation in order to link the process of geographical invasion to shifts in commensal and symbiotic microbiome communities, as such shifts may have dramatic impacts on the biology and/or vector competence of invading hematophagous insects.

## Introduction

Mosquitoes are considered by the World Health Organization to be the most medically important disease vectors. The Asian tiger mosquito (*Aedes albopictus*) is of major concern as it is known to be able to carry 26 arboviruses including Dengue and Chikungunya (Paupy et al., 2009). Furthermore, *Ae. albopictus* is considered as one of the most geographically invasive species. It has rapidly spread from its native area of South and East Asia to reach various eco-climatic regions in America, Africa, Oceania and Europe (Bonizzoni et al., 2013). The worldwide trades in secondhand tires and lucky bamboo, both of which often contain standing water making them ideal places for mosquito eggs and larvae, have been key factors in *Ae. albopictus* transportation. Once established in a new region, the tiger mosquito easily adapts and persists in a wide range of habitats, even in temperate climates mainly due to its aptitude to enter into a state of dormancy or “diapause” (Urbanski et al., 2010). Undoubtedly, the intrinsic capacities of the mosquito populations largely play an important role in their ecological plasticity. However, this assumption remains surprising as according to the “paradox of invasion,” recent introductions often imply a burden for the genetic structure of newly introduced populations (reviewed by Handley et al., 2011).

A comprehensive understanding of insect population genetics now requires an integrative approach considering microorganisms as a key component of the system. According to the hologenome theory, Metazoan organisms should no longer be considered as individuals, but rather as holobionts consisting of the host plus all of its associated microorganisms (Zilber-Rosenberg and Rosenberg, 2008). It is the holobiont and its associated hologenome that can be considered as a unit of selection which is impacted by variation, selection, drift and evolution (reviewed by Rosenberg and Zilber-Rosenberg, 2014). Insect holobionts are also difficult to decipher as they may include a range of host-symbiont relationships ranging from parasitism to mutualism (Toft and Andersson, 2010). Numerous studies have demonstrated the contribution of the microbiota to the biology of the host (Blottière et al., 2013; Douglas, 2014). Some mutualistic symbionts favor ecological adaptations in insects (Douglas, 2011, 2014) by playing key roles in extended phenotypes such as growth, nutrition, reproduction, protection against pathogens and tolerance to environmental stresses (Buchner, 1965; Dillon and Dillon, 2004; Moran et al., 2008; Moya et al., 2008). Moreover, the host genotype may also influence in return symbiont communities (Ochman et al., 2010; Muegge et al., 2011). It is thus, important to consider the genetic basis of bacterial microbiota selection.

It is striking that relatively few studies have focused on bacterial microbiota associated with invasive arthropods. Many invasive species harbor genetic modifications caused by founder effects or genetic drift, but the effect of such changes on their microbiota are only just beginning to be documented (Meusnier et al., 2001; Zurel et al., 2011; Ye et al., 2014). As symbiotic microorganisms can change more rapidly and by more diverse processes than the host organism itself they could influence the adaptation and evolution of the holobiont.

Here, we aimed to document whether populations from two representative areas colonized by the Asian tiger mosquito displayed changes in their host genotypes and associated bacterial microbiota. To that end, we sampled mosquitoes from field populations in Vietnam, a country located in the South East Asia where *Ae. albopictus* originated indicating ancient colonization, and in Metropolitan France, which was more recently invaded by this species. Mitochondrial and nuclear genotypes of mosquitoes and bacterial diversity were compared within and between populations.

## Materials and methods

### Sampling areas and mosquito collection

**Figure 1:**
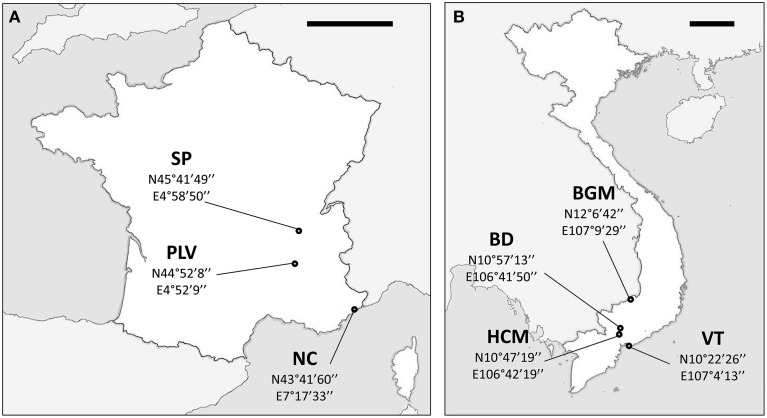
**Maps of sampling sites**. Sampling sites and GPS coordinates (*World Geodetic System 1984*) in France **(A)** and Vietnam **(B)**. NC, Nice; PLV, Porte-lès-Valence; SP, Saint Priest; VT, Vung Tàu City; HCM, Hồ Chí Minh City; BD, Bình Du'o'ng, BGM, Bù Gia Mâp. Scale bars, 200 km.

*Ae. albopictus* specimens were sampled in Metropolitan France and Vietnam. In addition to their contrasted climate and ecology, these two countries were chosen as Vietnam is located in the South East Asia, the region where *Ae. albopictus* originated indicating ancient colonization, whereas Metropolitan France is a newly invaded zone. Sampling in Metropolitan France was performed between August and September 2012 at Saint-Priest (SP), Portes-Lès-Valence (PLV), and Nice (NC). NC is one of the first invaded sites in France since 2004 (Medlock et al., 2012), whereas PLV and SP were colonized in 2011 and 2012, respectively (data obtained from French health organization INVS). Mosquito sampling in Vietnam was performed during October 2012 at Hồ Chí Minh City (HCM), Bình Du'o'ng (BD), Vung Tàu City (VT), and Bù Gia Mâp (BGM) (Figure 1). All sites were urban or suburban, except BGM located in a protected forest national park. Sampling sites were at least 18 km from each other to avoid sampling populations originated from the same breeding site. Consequently, we assume that individuals collected from the same sites belong to the same population as they share breeding sites, and a total of seven independent populations (three from Metropolitan France and four from Vietnam) were obtained and analyzed. Live adult females were caught with nets or BG-Traps and then identified using morphological characteristics (Rueda, 2004). For some individuals, identification was confirmed by COI barcode sequencing (see below). To control confounding effects from nutritional factors, only females that could be seen to contain no blood upon magnified observation of the gut contents were retained for analysis. Mosquitoes were stored in 100% ethanol at −80°C until used.

### Quantification of *wolbachia w*AlbA and *w*AlbB strains

*Wolbachia w*AlbA and *w*AlbB were quantified in whole bodies of 10 mosquitoes from each site (Table S1). Their densities were measured in triplicate by qPCR amplification of the *Wolbachia wsp* gene and normalized with the *Ae. albopictus actin* gene as described (Zouache et al., 2009). Standard curves were drawn on DNA plasmid pQuantAlb which contains *wsp* genes of *Wolbachia pipientis w*AlbA and *w*AlbB as well as *actin* gene of *Ae. albopictus* (Tortosa et al., 2008). Correlations between the two strains were calculated with R software using the Pearson's correlation.

### Sample preparation, miseq sequencing, quality trimming and diversity analysis of sequences

Previous work (Minard et al., 2014) showed that whole insect body was inappropriate for in-depth analysis of bacterial microbiota of *Ae. albopictus* by NGS due to the overrepresentation of *Wolbachia* sequences. Here we used midguts that are known to be a key organ in the metabolism and immunity of mosquitoes as well as the first point of entry for transmitted viruses (Clements, 1992; Jupatanakul et al., 2014; Kenney and Brault, 2014b). In addition this organ was shown to harbor a moderate density of *Wolbachia* in *Ae. albopictus* (Zouache et al., 2009).

Prior to dissection for midgut recovery from 32 individuals (Table S1), female specimens were surface-disinfected with 70% ethanol and rinsed with sterile water as previously described (Minard et al., 2013). All dissection steps were performed under a sterile laminar flow hood in a containment environment. Mosquitoes were dissected in sterile 1 × phosphate buffered saline solution (Life Technologies, NY, USA). For each mosquito, the midgut was separated from the rest of the body. Midguts were then individually crushed with 1-mm diameter beads in ATL lysis buffer (Qiagen, Hilden, Germany) containing 20 mg.ml^−1^ lysozyme (Euromedex, Strasbourg, France) using a Bioblock Scientific MM 2000 mill (Retsch, Eragny sur Oise, France). DNA was then extracted with Qiagen DNeasy Blood and Tissue kit (Qiagen, Hilden, Germany) following the manufacturer's recommendations for both Gram negative and Gram positive bacteria. Assuming that the remaining mosquito bodies (hereafter referred to as carcasses) should be dominated by *Wolbachia*, they were pooled per population (Table S1) and DNA extracted as above, and then used as positive controls. Finally, as negative controls to evaluate potential contamination, DNA extraction was carried out without any biological matrix and four independent eluates were concentrated and pooled.

Hypervariable V5-V6 *rrs* regions were amplified in triplicate for each DNA sample with 30 ng of DNA and modified primers 784F (5′-AGGATTAGATACCCTGGTA-3′) and 1061R (5′-CRRCACGAGCTGACGAC-3′) as described (Andersson et al., 2008) with modifications. Briefly, primers containing a 8-bp multiplex barcode and Illumina adapters were used for PCR amplifications with 1.75 U of Expand High Fidelity Enzyme Mix (Roche, Basel, Switzerland), 1 × Expand High Fidelity Buffer (Roche, Basel, Switzerland), 0.06 mg mL^−1^ of T4 gene 32 protein (New England Biolabs, Evry, France), 0.06 mg mL^−1^ of bovine serum albumin (New England Biolabs, Evry, France), 40 μM of dNTP mix, 200 nM of each primer (Life Technologies, Saint Aubin, France). Amplifications were carried out on Biorad C1000 thermal cycler (Biorad, CA, USA) with 5 min at 95°C, followed by 40 cycles at 95°C for 40 s, 54.2°C for 1 min, 72°C for 30 s, with a final extension step of 7 min at 72°C. Forty PCR amplification cycles were necessary to generate an optimal amount of amplicons for Miseq sequencing. The three PCR product replicates from each sample were pooled, purified with Agencourt AMPure XP PCR Purification kit (Beckman Coulter, Villepinte, France), and quantified using the Quant-iT Picogreen dsDNA Assay Kit (Life Technologies, NY, USA).

A total of 40 amplicon libraries were constructed: 32 for individual midguts, 7 for carcasses and 1 for the negative control. Sequencing of each library was performed on the Illumina MiSeq platform (2 × 250-bp paired-end reads) by ProfileXpert—ViroScan 3D (Lyon, France). All FastQ files were deposited at EMBL European Nucleotide Archive (https://www.ebi.ac.uk/ena) under the project accession number PRJEB6896. A total of 9, 222, 165 reads were obtained, paired-end reads were joined with PandaSeq (Masella et al., 2012), trimmed and aligned on the SILVA database release 115 using standard filtering tools in the MOTHUR pipeline (Schloss et al., 2009). Two errors were allowed in primer sequences, read sizes were filtered to be 200–350 bp in length with no ambiguous bases. Chimeras were detected and removed with Perseus implemented in Mothur package. Based on the analysis of clustered sequence rates from 92 to 99% similarity, OTUs were re-adjusted to 97% similarity using a median neighbor algorithm. Sequences were classified according to the SILVA database release 115 at 80% minimum bootstrap using a naïve Bayesian classifier (Wang et al., 2007). OTUs were also kept if there were at least represented by more than one sequence overall samples. Furthermore, OTUs were removed from further analyses if they were detected in the negative control sample and their relative abundance was not at least 10 times greater than that observed in the negative control. This additional quality control criterion allows us to qualify and correct for low concentration contaminants of experimental origin. Richness, α and β diversity indices were calculated using a subsample of the same read number for each sample. Diversity analyses, hierarchical analysis of molecular variance (AMOVA), Non-Metric Multidimensional Scaling ordination and heatmap representations were performed with R software (R Development Core Team, 2009) using ade4 and vegan packages (Dray and Dufour, 2007; Oksanen et al., 2013). To highlight possible country-associated OTUs, extended errors bars were computed and classified according to Welch modified *t*-test significance (*p* < 0.05) using STAMP software 2.0.9 (Parks and Beiko, 2010).

### Mitochondrial gene amplification and haplotyping

To maximize the quality and quantity of DNA obtained, our previously optimized extraction protocol for individual whole mosquito was used (Minard et al., 2014). A total of 85 individuals were analyzed ranging from 9 to 20 individuals per sampling site (Table S1). The 597-bp region of mtDNA cytochrome c oxidase subunit I (*COI*) gene was amplified with CI-J-1632 (5′-TGATCAAATTTATAAT-3′) and CI-N-2191 (5′-GGTAAAATTAAAATATAAACTTC-3′) primers using 45 ng of DNA matrix as described (Raharimalala et al., 2012). To analyze haplotypes*, COI* sequences were aligned with Seaview 4, then diversity and nucleotide composition were calculated with DnaSP (Librado and Rozas, 2009). AMOVA statistical analyses were performed with Arlequin 3.5 × (Excoffier and Lischer, 2010). Sequences of the different *COI* haplotypes were deposited on Genbank (https://www.ncbi.nlm.nih.gov/genbank) under the accession numbers LM999972-LM999977.

### Microsatellite processing and genotyping

A total of 199 individuals were genotyped ranging from 22 to 30 individuals per sampling site (Table S1). Amplifications were done with 10 ng of DNA extracted from each individual and master mix from Qiagen Type-it Microsatellite PCR Kit following the manufacturer's recommendations (Qiagen, Hilden, Germany). Amplifications were performed on Biorad C1000 thermal cycler (Biorad, CA, USA) with an optimal protocol for each microsatellite to minimize unspecific artifacts. A set of 11 microsatellite markers previously described (Chambers et al., 1986; Porretta et al., 2006; Beebe et al., 2013) were used; namely AealbA9, AealbB51, AealbB52, AEDC, Alb-di6, Alb-tri3, Alb-tri18, Alb-tri25, Alb-tri41, Alb-tri45, and Alb-tri6 (Table S2). For AeablA9, AealbB51, and AealbB52 microsatellites, the program consisted of 5 min at 94°C, followed by 35 amplification cycles at 94°C for 5 min, 52°C (AealbA9) or 50°C (AealbB51, Aealb0B52) for 30 s and 72°C for 45 s, with a final step of 30 min at 72°C. AEDC microsatellite sequences were amplified with 5 min at 94°C followed by 30 cycles of 45 s at 94°C, 1 min 30 s at 56°C and 45 s at 72°C, with a final step of 30 min at 60°C. Alb-di6, Alb-tri3, Alb-tri18, Alb-tri25, Alb-tri41, Alb-tri45, and Alb-tri6 were amplified as described by Beebe et al. (2013). PCR products were diluted (between 1/60 and 1/100 depending on the relative sensitivity of markers) then 1 μL was mixed with 13.8 μL of ultrapure Hi-Di-formamide TM and 0.2 μL of size marker (MRL 500) and loaded on an ABI Prism 3730XL Genetic Analyzer automated sequencer (Life Technologies, NY, USA). Microsatellites were scored manually with Genemapper 3.0 (LifeTechnologies, NY, USA). Null alleles were evaluated with FreeNA (Chapuis and Estoup, 2007). Diversity indices, linkage disequilibrium, Factorial Correspondence Analysis and hierarchical AMOVA analyses were computed with Genetix 4.05, Fstat 2.9.3.2 and Arlequin 3.5x softwares (Excoffier and Lischer, 2010). The Bayesian structure of populations was evaluated using Structure 2.3.4 (Pritchard et al., 2000) with 100 000 “burn-in” steps followed by 500 000 iterations. Runs from 1 to 8 potential groups (K) were processed with 20 replicates (Figure S1). An admixture model was used with a location prior factor. As recommended for datasets with possible null alleles, a dominant allele option was set in the model. The best fit K-value was chosen with the Evanno method implemented in STRUCTURE HARVESTER (Evanno et al., 2005; Earl and VonHoldt, 2012) and the 20 replicates were averaged with CLUMPP (Jakobsson and Rosenberg, 2007). Finally, a population structure barplot was drawn with DISTRUCT (Rosenberg, 2004). Comparisons between Fst distances and bacterial microbiota Bray-Curtis distances were performed with a Mantel test. Rarefied genetic richness (Ar) and diversity (Hs) were correlated with Shannon bacterial diversity using Spearman's rank correlation. As populations which experienced a recent reduction of their effective size can develop a heterozygosity excess at neutral loci, this parameter was tested using BOTTLENECK software (Cornuet and Luikart, 1996).

## Results

### *wolbachia w*AlbA and *w*AlbB strains are abundant and positively correlated with each other

**Figure 2:**
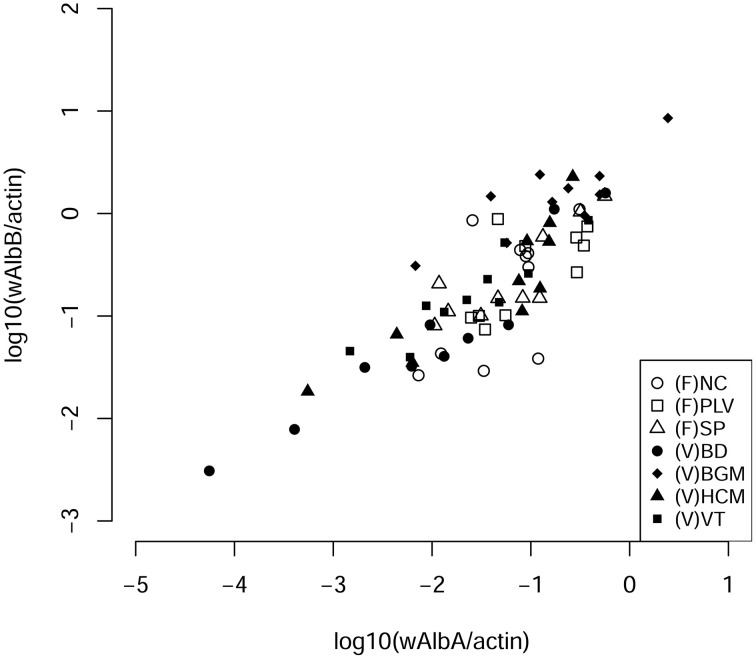
**Correlation densities of ***Wolbachia pipientis*****. The number of each bacterial strain per cells was evaluated by quantification of *wsp* genes from each *Wolbachia w*AlbA and *w*AlbB strains normalized to the number of host actin gene copies (Pearson's correlation *R*^2^ = 0.84, *p* < 2.2 × 10^−16^). NC, Nice; PLV, Porte-lès-Valence; SP, Saint Priest; VT, Vung Tàu City; HCM, Hồ Chí Minh City; BD, Bình Du'o'ng, BGM, Bù Gia Mâp.

**Figure 3:**
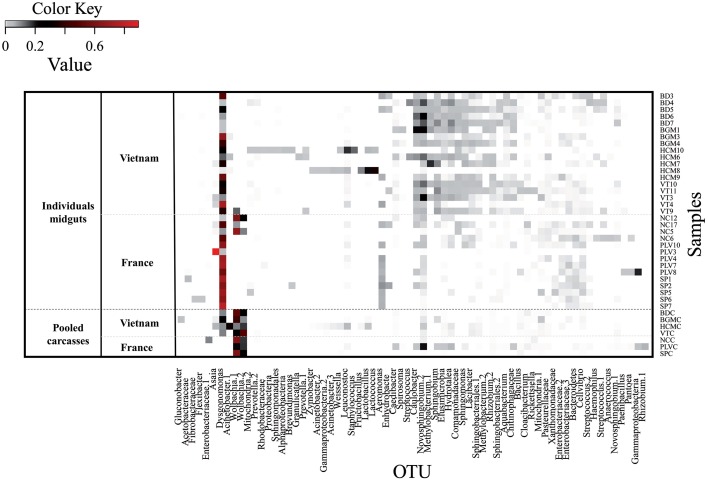
*****Aedes albopictus*** microbiota bacterial diversity**. The heatmap represents the relative abundance of 3% OTUs in each sample. The Color Key Value refers to the proportion of an OTU in a single sample. The OTUs were represented if their proportion was =0.01 in at least one sample. They were named by their taxonomic assignment according to Bayesian classification. When more than one OTU get the same assignation, they were differentiated with numbers. Samples are classified according to their own populations. Individual midgut samples from Vietnam were collected in Bình Du'o'ng (BD 3, 4, 5, 6, and 7), Bù Gia Mâp (BGM 1, 3, 4), Hồ Chí Minh City (HCM 6, 7, 8, 9, 10), Vung Tàu City (VT 3, 4, 9, 10, 11). Individual midgut samples from France were collected in Nice (NC 5, 6, 12, 17), Portes-Lès-Valence (PLV 3, 4, 7, 8, 10), and Saint Priest (SP 1, 2, 5, 6, 7). The pooled carcasses of the following individuals were also analyzed for both Vietnamese (BDC, BGMC, HCMC, VTC) and French populations (NCC, PLVC, SPC); the last “C” added to each name indicates carcass.

Our aim was to study the bacterial microbiota in autochthonous and invasive tiger mosquito populations. In a previous study, we demonstrated that *Wolbachia* is the predominant bacterial species in *Ae. albopictus* from Madagascar when using whole body genomic DNA, constituting up to 99% of high throughput sequences recovered (Minard et al., 2014). Here, we first tested whether the two *Wolbachia* strains *w*AlbA and *w*AlbB were also present and dominant in mosquitoes sampled from autochthonous populations at four sites (HCM, BD, VT, BGM) in Vietnam and from invasive populations at three sites (SP, PLV, NC) in France (Figure 1). *w*AlbA and *w*AlbB were detected in all individuals of the seven populations. The lowest densities of *w*AlbA and *w*AlbB strains detected were 5.25 × 10^−5^ wsp.actin^−1^ and 3.09 × 10^−3^ wsp.actin^−1^ copies respectively in mosquito samples from BD and the highest densities of 2.44 wsp.actin^−1^ and 8.53 wsp.actin^−1^ copies respectively from the BGM population (Figure 2). A significant positive correlation (Pearson's correlation *R*^2^ = 0.84, *p* < 2.2 × 10^−16^) between *w*AlbA and *w*AlbB strains was found among all the populations tested (Figure 2). To check if *Wolbachia* sequences were overrepresented in bacterial microbiota sequences when applying NGS methods to the whole body, the V5-V6 *rrs* amplicons were generated from mosquito carcass pools as indicated in material and methods, and sequenced by Miseq technology. Results confirmed a dominance of *Wolbachia* OTUs (Figure 3) which account for 28% (for HCM) to 91% (for SP) of the sequence dataset, reinforcing the rationale for our choice to avoid using the whole insect body for bacterial community analysis in *Ae. albopictus*.

### Midgut bacterial community structure in vietnamese autochthonous populations compared to french invasive ones

**Figure 4:**
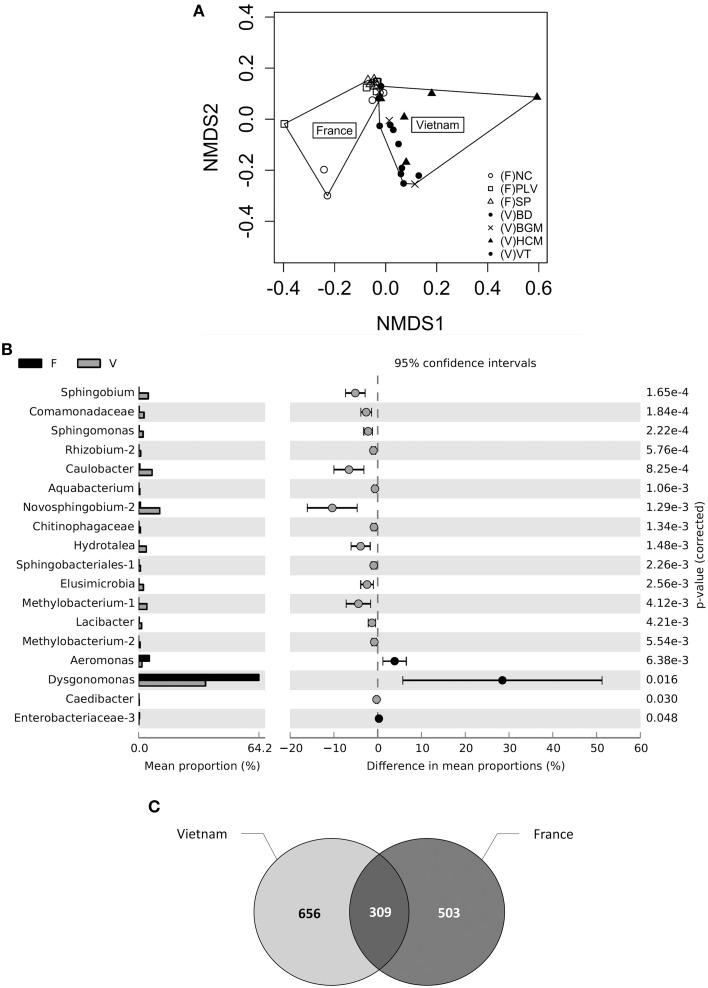
**Community structure and OTU-country associations**. **(A)** Non-Metric Multidimentional Scaling plot represents Bray-Curtis β-diversity structure among individuals and populations. The loss function Stress = 0.09 and correlation with true distances *R*^2^ = 0.97. NC, Nice; PLV, Porte-lès-Valence; SP, Saint Priest; VT, Vung Tàu City; HCM, Hồ Chí Minh City; BD, Bình Du'o'ng; BGM, Bù Gia Mâp. **(B)** Difference in OTU abundance between midgut bacterial microbiota of French and Vietnamese *Ae. albopictus*. Display represents extended error bars of significant fold-changes (*P*>0.05) between midgut samples from France (F) and Vietnam (V). **(C)** Venn diagram representing shared OTUs between midgut samples from France and Vietnam. The intersection of both circles represents the number of shared OTUs between France and Vietnam. To avoid size effect, sequences were merged per group, and then subsampled.

The insect gut is a key organ in insect physiology and immunity. Moreover, previous studies have demonstrated that this organ harbored low concentration of *Wolbachia* in *Ae. albopictus* adults (Zouache et al., 2009), opening up the possibility to extend the depth of analysis of the gut-associated microbial community. For this purpose, V5-V6 *rrs* amplicons from 32 individual midgut samples (from 3 to 5 individuals per sampling site) were sequenced with MiSeq technology. Analysis of a negative control showed the presence of bacterial sequences that probably derived from contamination during laboratory sample handling (Table S6). However, the diversity of this control was dissimilar from those of all mosquito samples (Bray-Curtis dissimilarity > 68.6%). For subsequent analysis of sequences associated with mosquito samples, OTUs potentially originating from laboratory contamination were trimmed from the whole dataset. Based on this analysis, a total of 2,088 OTUs were identified in all the midgut samples (between 306 and 1,272 OTUs per sample), with a total of 68 OTUs exceeding 1% in abundance. These OTU numbers were consistent with those previously obtained by high throughput sequencing of midguts from various mosquito species (Osei-Poku et al., 2012). The genus *Dysgonomonas* was the most prevalent and abundant OTU retrieved from the midgut samples (Figure S3), although its abundance varied from 3% (HCM8) to 72% (SP7) between samples (Figure 3). AMOVA analysis and ordinations were performed to detect the degree of differentiation at various hierarchical levels. No significant variation was observed between sites, indicating a low variability between individuals belonging to a given population. In contrast, variation between countries was found to be significant for the β-diversity measure, explaining a large part of the variation for Bray-Curtis dissimilarities (AMOVA, 22.79%, *p* < 10^−4^) (Table 1, Figure 4A). Similar structures were obtained with Unifrac phylogeny based β-diversity distances (Table S3). Consequently, further comparisons of populations were performed at the country level. When compared with the four populations from Vietnam, consisting of a total of 14 individuals, the three populations from France composed of 18 individuals harbored less diverse and more homogeneous bacterial microbiota, indicated by lower values for the Chao 1 richness estimator (Mann–Whitney Wilcoxon, *p* = 0.002), Shannon-Weather α-diversity (Mann–Whitney Wilcoxon, *p* < 10^−3^) and variance of abundance-weighted β-diversity (Figure S2). However, Bray-Curtis (Beta-dispersion, *p* = 0.33) distances were not significantly different between populations of the two countries.

**Table 1:** **AMOVA analysis**.

	**Haplotypes**			**Microsatellites**			**β-Diversity (Bray-Curtis)**		
	df^*^	**Variance (%)**	***p***	df^*^	**Variance (%)**	***p***	df^*^	**Variance (%)**	***p***
Among countries	1	54.3	0.02	1	2.61	0.2	1	22.79	< 10^−4^
Among populations/Countries	5	20.54	< 10^−3^	5	12.61	< 10^−3^	5	7.14	0.48
Within populations	78	25.15		391	84.78		26	70.07	

* *df, degree of freedom*.

Overall, 15 OTUs were shown to be significantly associated with Vietnamese populations (Welch corrected *t*-test, *p* < 0.05) (Figure 4B), including various members of *Shingomonadaceae* family (*Sphingobium, Novosphingobium, Sphingomonas*). Only three OTUs assigned to *Dysgonomonas* and *Aeromonas* genera and *Enterobacteriaceae* family were shown to be significantly associated with French populations. The OTU overlap was calculated after merging and subsampling sequences at the country level (Figure 4C). The resulting Venn diagram showed 21% of shared OTUs (*n* = 309), and 34% (*n* = 503), and 45% (*n* = 656) specific of France and Vietnam, respectively. Shared OTUs counted for 85% of the overall sequences.

### Low mitochondrial DNA variation among *Ae. albopictus* populations from france and vietnam

The genetic makeup of *Ae. albopictus* hosts was evaluated by barcoding based on mitochondrial *COI* gene sequences. A total of 6 haplotype variants were found in individuals from all populations. The genetic heterogeneity was more pronounced between countries (AMOVA 47.4%, *p* < 10^−3^) than between sites (AMOVA 19.79%, *p* < 10^−3^) (Table 1). The two major subclades were distinguishable by only one mutation. One subclade included 52 haplotypes (H_1) and the other 28 haplotypes (H_3) (Table S4). H_1 was mostly associated with the populations from Vietnam whereas H_3 was more associated with those from France. However, a mix of both haplotypes was also found in the populations at the PLV site in France (12/20 for H_1 and 7/20 for H_3) and at the BD site in Vietnam (16/17 for H_1 and 1/17 for H_3) (Figure S4).

### Evidence for genetic reduction in *Ae. albopictus* populations invasive to france

**Figure 5:**
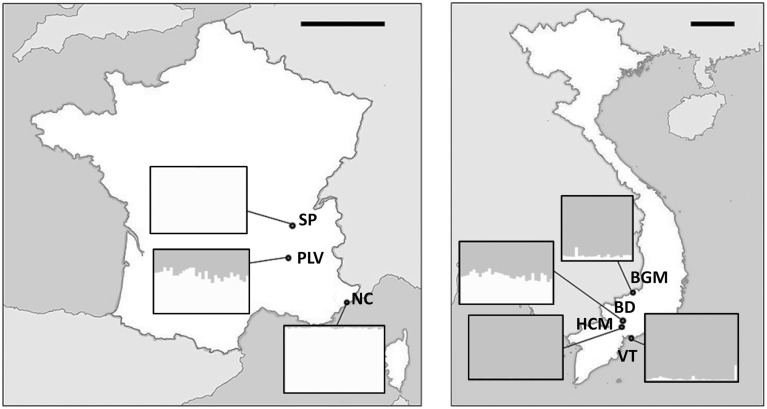
**Genetic structures of ***Aedes albopictus*** populations**. The map of the microsatellite genetic structure (*K* = 2) for each site. Each bar represents an individual and the grayscale represents the probability that an individual belongs to a population. Scale bar of the maps, 200 km. NC, Nice; PLV, Porte-lès-Valence; SP, Saint Priest; VT, Vung Tàu City; HCM, Hồ Chí Minh City; BD, Bình Du'o'ng; BGM, Bù Gia Mâp.

The mosquito nuclear genomic variation was characterized further by genotyping 199 individuals with 11 microsatellite markers. The overall number of alleles per locus varied from 6 (AealbB52) to 30 (Alb-tri 18). Rarefied allele richness of populations from each site was 4.56 for SP, 4.57 for PLV, 5.91 for NC, 6.50 for HCM, 7.47 for VT, 8.25 for BGM, and 8.62 for BD. To test for hypothetical population bottlenecks or expansions, the allelic richness (*Ar*) and the heterozygosity (He) at each genomic locus were analyzed for each sampling site. For both these analyses of variation, values for populations from France were significantly lower than those from Vietnam (Mann–Whitney, *p* = 0.0003 and 0.0003, respectively). The bottleneck analysis did not show any significant heterozygosity excess under a two-phase model or a single stepwise model (Table S5). No significant linkage disequilibrium was found between any pair of loci. All populations had a positive inbreeding index (F_IS_) between 0.12 (for PLV) and 0.196 (for HCM) reflecting an excess of homozygotes (Table 2). Moreover, there were significant scores for the presence of null alleles. However, F_ST_ values of the entire mosquito sample were 0.142 (CI_95%_ 0.058–0.269) with ENA correction for null alleles and 0.145 (CI_95%_ 0.061–0.270) without correction. Consequently, the presence of null alleles did not strongly impact the estimation of differentiation. The structure of the populations was evaluated with the Bayesian method of assignment. The optimal number of clusters selected with the second-order change in likelihood method was *K* = 2 (Figure S1) (Evanno et al., 2005). Populations were clustered in two different genetic groups according to country of origin, except for populations from PLV in France and BD in Vietnam, which harbored a mixture of both genotypes (Figure 5, Figure S5). AMOVA analysis revealed a non-significant variation in the structure among countries (AMOVA 2.6%, *p* = 0.2) but a moderate variation among sites within a country (AMOVA 13%, *p* < 10^−3^) (Table 1).

**Table 2:** **Microsatellite characteristics among populations**.

**Country**	**Site**	**Index**	**di-6**	**tri-3**	**tri-18**	**tri-25**	**tri-41**	**tri-45**	**tri-6**	**B51**	**B52**	**A9**	**AEDC**	**All**
Vietnam	BD	Nall	0.03	**0.21**	0.03	**0.16**	0	0.07	0	0	0	0.01	0	0.046
		Fis	0.114	0.49	0.125	0.366	0.058	0.211	0.038	−0.036	−0.14	−0.008	−0.164	0.133
		He	0.812	0.828	0.92	0.798	0.834	0.746	0.852	0.95	0.259	0.813	0.593	0.717
		Ho	0.733	0.433	0.82	0.519	0.8	0.6	0.833	1	0.3	0.833	0.7	0.636
	HCM	Nall	0	**0.17**	0.01	**0.22**	0.11	0.05	0.04	0.06	0	0.06	0.03	0.068
		Fis	−0.105	0.395	0.183	0.556	0.533	0.109	0.098	0.116	na	0.188	0.137	0.196
		He	0.683	0.723	0.68	0.691	0.723	0.882	0.798	0.633	0	0.624	0.493	0.659
		Ho	0.767	0.448	0.57	0.318	0.533	0.8	0.733	0.645	0	0.517	0.433	0.537
	VT	Nall	0	0.01	**0.16**	**0.25**	**0.13**	0	0	0	0	0.07	0	0.056
		Fis	−0.137	0.146	0.36	0.588	0.305	0.049	−0.008	na	−0.061	0.123	−0.309	0.122
		He	0.77	0.696	0.87	0.725	0.803	0.848	0.866	0	0.364	0.827	0.647	0.706
		Ho	0.889	0.607	0.57	0.308	0.571	0.821	0.889	0	0.393	0.741	0.857	0.615
	BGM	Nall	0	**0.19**	0.01	0.02	**0.08**	**0.13**	**0.11**	0.05	**0.12**	**0.16**	0.01	0.08
		Fis	0.026	0.429	0.012	0.071	0.218	0.267	0.264	0.119	0.267	0.369	−0.309	0.171
		He	0.775	0.693	0.9	0.7	0.791	0.783	0.84	0.386	0.542	0.838	0.65	0.729
		Ho	0.773	0.409	0.91	0.667	0.636	0.591	0.636	0.35	0.41	0.546	0.86	0.627
France	NC	Nall	0.08	0.21	0.07	0.03	0	0.2	**0.09**	0	0	0.07	0	0.068
		Fis	0.129	0.562	0.328	0.017	−0.057	0.539	0.223	na	−0.024	0.136	−0.141	0.181
		He	0.638	0.634	0.72	0.756	0.807	0.655	0.825	0	0.099	0.847	0.654	0.642
		Ho	0.567	0.286	0.5	0.762	0.867	0.31	0.655	0	1.03	0.75	0.762	0.545
	PLV	Nall	**0.08**	**0.13**	0.06	**0.12**	0.01	0	0.05	0	**0.11**	**0.22**	0	0.071
		Fis	0.207	0.507	0.108	0.211	0.033	−0.143	0.13	na	0.487	0.51	−0.306	0.152
		He	0.494	0.292	0.77	0.727	0.745	0.316	0.827	0	0.127	0.686	0.504	0.551
		Ho	0.4	0.148	0.7	0.607	0.733	0.367	0.733	0	0.067	0.345	0.667	0.475
	SP	Nall	0.04	0.03	0	0.09	0.05	0.01	0.08	0	0	0.05	0.06	0.037
		Fis	0.005	0.154	−0.012	0.223	0.122	0.011	0.191	na	na	0.2	0.176	0.12
		He	0.579	0.4	0.84	0.765	0.757	0.48	0.71	0	0	0.675	0.574	0.706
		Ho	0.586	0.345	0.86	0.607	0.679	0.483	0.586	0	0	0.552	0.483	0.615

*NC, Nice; PLV, Porte-lès-Valence; SP, Saint Priest; VT, Vung Tàu city; HCM, Hồ Chí Minh City; BD, Bình Du'o'ng, BGM, Bù Gia Mâp; Nall, null alleles frequency; Fis, fixation index; He, expected heterozygosity; Ho, observed heterozygosity. Significant frequencies of null alleles are in bold*.

### Positive correlation between bacterial and genetic diversities of *Ae. albopictus* populations

**Figure 6:**
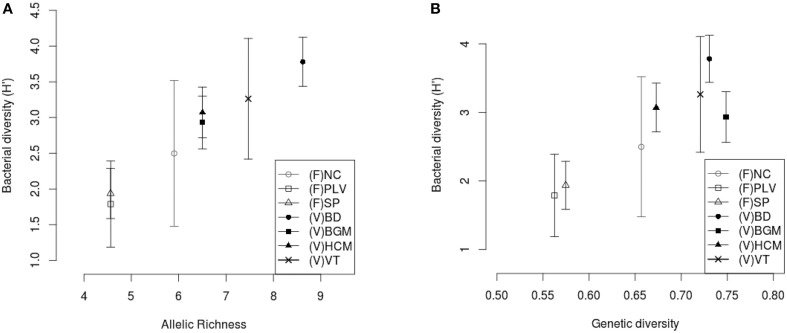
**Correlation between host genetic richness, host genetic diversity, and midgut bacterial diversity**. The mean bacterial Shannon α-diversity (H′) was correlated with **(A)** rarefied genetic richness, Ar (Spearman's rank correlation, ρ = 0.95, *p* = 8.10^−3^) and **(B)** diversity, Hs (Spearman's rank correlation, ρ = 0.78, *p* = 0.048). (F), France; (V), Vietnam; NC, Nice; PLV, Porte-lès-Valence; SP, Saint Priest; VT, Vung Tàu City; HCM, Hồ Chí Minh City; BD, Bình Du'o'ng; BGM, Bù Gia Mâp. Standard deviation of Shannon α-diversity (H′) was represented for each site.

The populations sampled were compared to assess whether there was any relationship between the bacterial diversity and the genetic diversity of the mosquitoes. Comparative analysis showed a low correlation between bacterial β-diversity (Bray-Curtis dissimilarity distance) and haplotype structure (Mantel, *R*^2^ = 0.5, *p* = 0.02) and no significant correlation with the Cavalli-Sforza Edwards measure of microsatellite genetic distance (Mantel, *R*^2^ = 0.20, *p* = 0.19). However, for all sampling sites, positive correlations were observed between mean bacterial α-diversity (*H*′) and respectively host *Ar* (Spearman's rank correlation, ρ = 0.95, *p* = 8.10^−3^) (Figure 6A) and host genetic diversity (*Hs*) (Spearman's rank correlation, ρ = 0.78, *p* = 0.048) (Figure 6B).

## Discussion

Mosquitoes may be regarded as holobiont units in which the host and its microbiota may display symbiotic relationships and multitrophic interactions (Minard et al., 2013). As demonstrated in other insect models such as drosophila and aphids (Engel and Moran, 2013; Lizé et al., 2014), it is envisaged that mosquito-associated bacterial microbiota could influence the ability of the host to respond to biotic and abiotic factors. An essential step forward is to increase our knowledge on the mosquito-associated microbiota. Here, we used a next generation sequencing method and metabarcoding to characterize the composition of bacterial microbiota of invasive and autochthonous populations of *Ae. albopictus* and to test for correlations with host genotype.

*Wolbachia* is the most studied bacterium of mosquitoes. Although, some beneficial fitness effects of this endosymbiont have been demonstrated in *Ae. albopictus* (Dobson et al., 2002), *Wolbachia* most commonly alters mosquito reproduction by inducing cytoplasmic incompatibility between infected males and uninfected females (Stouthamer et al., 1999). Superinfection with more than one *Wolbachia* strain makes the system more complex and some models predict that superinfected females have a selective advantage as they should be able to reproduce with every combination of uninfected and strain-infected males (Dobson et al., 2004; Tortosa et al., 2010). Diagnostic PCR on field-caught *Ae. albopictus* from various countries confirmed this prediction as more than 99% of individuals were superinfected with *Wolbachia w*AlbA and *w*AlbB strains (Kittayapong et al., 2002; de Albuquerque et al., 2011; Zouache et al., 2011). Here we showed a 100% prevalence of such double infection in seven *Ae. albopictus* populations, three from France and four from Vietnam. Other than cytoplasmic incompatibility and the age of the mosquito (Tortosa et al., 2010), factors driving *Wolbachia* persistence in *Ae. albopictus* remain largely unknown. Our results demonstrated for the first time a strong correlation between the proportions of the two strains *w*AlbA and *w*AlbB in *Ae. albopictus* natural populations, suggesting a structured mechanism is regulating their co-occurrence. *Wolbachia* has also been demonstrated to induce selective “sweep” on mitochondria, which drastically reduces the genomic variability of this organelle in *Ae. albopictus* (Hurst and Jiggins, 2005). Accordingly, the mitochondrial haplotypes of all seven populations showed very low nucleotide diversity in the *COI* gene (haplotypes differed by only one substitution) and low richness (less than three haplotypes per sampling site after rarefaction). Therefore, mitochondrial markers are highly sensitive to admixture in comparison with nuclear markers such as microsatellites. For this reason, it is not suitable to rely on mitochondrial haplotypes when estimating intraspecific genetic diversity in *Ae. albopictus*.

As confirmed herein, analyses of the *Ae. albopictus* microbiota are systematically dominated by *Wolbachia* sequences (Minard et al., 2014). In order to investigate other genera we used the midgut tissue, which is recognized to be poorly colonized by this bacterium (Zouache et al., 2009). Moreover, in arthropod vectors, this organ is the main site for multipartite interactions between bacterial microbiota, arboviruses and the host (Jupatanakul et al., 2014; Kenney and Brault, 2014a). Interestingly, 21% of total OTUs richness was found in populations from both France and Vietnam, assuming that some members of the microbiota can be shared among *Ae*. *albopictus* from contrasted populations. Despite the relatively low richness of shared OTUs, they accounted for 85.2% of all the sequences. The dominance of the shared microbiota over the transient microbiota is suggestive of positive selection of the interactions that constitute semi-constant gut microbiota (Figure S3), in contrast with the variable microbiota previously described within midguts of field-caught mosquitoes belonging to different species (Osei-Poku et al., 2012). Although, this observation may also be explained by the relatively homogeneity of the habitats of *Aedes* spp. mosquitoes (low oxygen pressure, a pH comprised between 8 and 10) (Dillon and Dillon, 2004; del Pilar Corena et al., 2005; Saboia-Vahia et al., 2014), and the establishment of habitat-specific bacterial associations. However, some factors such as the type of nutrients ingested and temperature (not regulated in ectotherms) can strongly vary between French and Vietnamese populations. In addition, previous studies highlighted the importance of water of breeding sites in which mosquito larvae and pupae develop. It was shown that mosquitoes acquire a large part of their microbiota from larval stages which themselves depend on the bacterial composition of the water of breeding sites. Moreover, a great variability in diversity of abundance of taxa was shown between stages according to mosquito species (Pumpuni et al., 1996; Minard et al., 2013; Coon et al., 2014; Dada et al., 2014; Gimonneau et al., 2014). Following these observations, the variations observed among the populations studied here could be linked with environmental factors of their habitat.

Among all the midgut samples, a total of 68 dominant OTUs were described and assigned with the name of their most probable phylotype. *Dysgonomonas* was the most prevalent and abundant one. This genus belongs to the phylum *Bacteroidetes*, and has already been recently detected in *Ae. albopictus* from Madagascar (Minard et al., 2014). Prevalent and abundant bacteria belonging to the *Bacteroidetes* phylum were previously described in *Ae. aegypti*. In particular, this mosquito species harbors a high relative abundance of *Chryseobacterium* that is maintained during all different life stages of lab-reared populations (Coon et al., 2014). However, previous studies highlighted that gut mosquito microbiota is largely dominated by *Proteobacteria* as for *Anopheles coluzzi, An. funestus, An. gambiae, An. Stephensi*, and *Culex tarsalis* (Pidiyar et al., 2004; Lindh et al., 2005; Rani et al., 2009; Boissière et al., 2012; Minard et al., 2013; Gimonneau et al., 2014). For these studies one to two major genera were usually found dominant in the midgut, albeit the identity of a given dominant entity changes over individuals or populations (Boissière et al., 2012; Osei-Poku et al., 2012). Therefore, it is surprising that we describe a single prevalent and abundant taxon, *Dysgonomonas*, in the midgut of all individuals even originated from distantly separated populations, suggesting an evolutionary process that maintains the presence of this particular taxon. Interestingly, *Dysgonomonas* has also been identified in the gut of different animals such as termites (*Coptotermes formosanus, Macrotermes barneyi*), house flies (*Musca domestica*), fruit flies (*Drosophila* spp.), red palm weevils (*Rhynchophorus ferrugineus*), and sea bass (*Dicentrarchus labrax*) (Husseneder et al., 2009; Chandler et al., 2011; Carda-Diéguez et al., 2014; Tagliavia et al., 2014; Yang et al., 2014). This genus has been previously detected in *Anopheles stephensi* and *Culex tarsalis* microbiota with a moderate abundance (Rani et al., 2009; Duguma et al., 2013). Its ability to cause lysis of erythrocytes and to synthesize B_12_ vitamins might be a selective mechanism involved in a mutualistic interaction with female mosquitoes (Hironaga et al., 2008; Husseneder et al., 2009; Lawson et al., 2010; Yang et al., 2014). Some species of *Dysgonomonas* are both aerobic and facultative anaerobic, which could partly explain how some may adapt to the nearly anoxic insect midgut habitat (Johnson and Barbehenn, 2000; Chouaia et al., 2014). All these observations point to *Dysgonomonas* having a role in mosquito biology. Finally, certain *Dysgonomonas* species were also identified as human opportunistic pathogens, raising concern about the possibility of additional mosquito borne diseases and thus highlighting the recent concept of “pathobiome” in arthropod vectors (Hironaga et al., 2008; Lawson et al., 2010; Vayssier-Taussat et al., 2014).

Other bacterial genera detected in *Ae. albopictus* belong to the *Sphingomonadaceae* family (*Sphingomonas, Sphingobium, Novosphingobium*) and were preferentially associated with the autochthonous populations from Vietnam. These bacterial genera are ubiquitous in the environment (Vaz-Moreira et al., 2011; Ashton Acton, 2012) and are also able to colonize a variety of higher organisms (D'Auria et al., 2013; Zhang et al., 2013; Dai et al., 2014). They display various catabolic abilities including the production of hydrolases involved in the degradation of oligosaccharides, and in termite hosts they may participate in the degradation of plant compounds (Aylward et al., 2013). Moreover, these bacteria have already been identified in other plant-feeding insects including mosquitoes *Anopheles maculipenis, Anopheles gambiae, Anopheles stephensis*, and *Aedes aegypti* (Dong et al., 2009; Ramírez-Puebla et al., 2010; Dinparast Djadid et al., 2011; Gayatri Priya et al., 2012; Terenius et al., 2012; Koroiva et al., 2013). From these observations, it could be assumed that *Sphingomonadaceae* are important in making plant sugar available to the mosquito host, by degrading oligosaccharides in the mosquito gut or acquiring them from the environment.

Interestingly, the bacterial communities associated with the invasive mosquito populations in France were less diverse and more homogeneous than those associated with autochthonous populations in Vietnam. The living host environment is an important factor impacting the microbiota of insects (Linnenbrink et al., 2013). In mosquitoes, it is known that feeding behavior may drastically modify the structure of gut microbiota and strongly increase inter-individual variation (Wang et al., 2011; Pernice et al., 2014). To avoid the effects of short-term changes in midgut microbiota, we only studied unfed mosquitoes, but long-term feeding effects cannot be ignored. Indeed, mosquito nutrition is mostly based on nectar and *Ae*. *albopictus* is known to have a wide nutritional spectrum (Clements, 1992). Interestingly, previous studies of the microflora diversity of different environments highlighted a significant reduction of species richness within the flowering plants (angiosperms) in temperate compared to tropical ecosystems (Francis and Currie, 2003). Therefore, the diversity and availability of plant nutrient sources could explain the reduction and the homogeneity in bacterial diversity we observed in mosquito populations of France. Populations from France also harbor a lower genetic diversity. Genetic reductions in invasive species have been widely documented. The most probable factor explaining genetic reduction in invasive populations is that recent colonization by a reduced effective population size causes a founder effect bottleneck and genetic drift (Dlugosch and Parker, 2008). However, no evidence for a recent founder effect was detected in the invasive populations in France. The short generation time of *Ae*. *albopictus* as well as the history of complex and multiple introductions (evidenced especially in Portes-Lès-Valence populations which harbor low allelic richness and genetic diversity but an admixture structure discovered with both haplotype and microsatellite markers) may have erased signs of a past bottleneck. In addition, genetic diversity reductions in France could also be explained by other patterns (e.g. Landscape fragmentation, lowest effective size…). Interestingly, the most genetic diversified of these populations was in Nice, a invaded site since 2004 (Medlock et al., 2012), whereas Portes-Lès-Valence and Saint-Priest were colonized much later. As already suggested in various models (Sommer, 2005), genetic diversity reduction identified with neutral markers could be linked with diversity reduction of genes involved in immunity. Moreover, immune genes could also be under considerable selective pressure that would affect the composition of mosquito microbiota (Wang et al., 2011; Minard et al., 2013). Indeed, gut microbiota are involved in a strong reciprocal interaction with the host immune system in the mosquito gut (Hillyer, 2010; Cirimotich et al., 2011; Weiss and Aksoy, 2011). However, we did not find any correlations between microbiota and mosquito genetic structure based on neutral microsatellite markers. Interestingly, a genetic study of mice using quantitative markers demonstrated that a core microbiome was regulated by a complex polygenic trait likely to involve pleiotropic effects (Benson et al., 2010). However, as a direct link between genetic and microbial structures cannot be proven following our sampling design, further investigations would be necessary. In particular, development of *Ae. albopictus* quantitative markers would be helpful in pinpointing which host genetic factors could partly shape the microbiota diversity.

Reduction in diversity for both the mosquito host and its associated bacterial microbiota also raises questions about the possible impact on human pathogen transmission. During transmission cycles, mosquito-vectored pathogens pass through the host midgut epithelial membrane to reach hemolymph and salivary glands. This checkpoint is critical for transmission as the pathogen faces the microbiota barrier and its potentially antagonistic activity (enzymes, toxins, etc.) as well as the host immune system (Cirimotich et al., 2011; Weiss and Aksoy, 2011; Wang et al., 2013). Indeed, the microbiota of mosquito vectors was shown to interfere, positively or negatively, with their susceptibility to pathogen infection and transmission capacity (Dennison et al., 2014). For instance, a recent study on *Anopheles gambiae* mosquitoes suggested that reduction of gut microbiota diversity following ingestion of antibiotics increases the capacity of females to transmit *Plasmodium falciparum* (Gendrin et al., 2015). In previous works it was demonstrated that a high proportion of viral particles were able to disseminate beyond the insect midgut barrier in *Ae. albopictus* populations from Cagne-Sur-Mer (~12 km away from Nice in France) (Vega-Rua et al., 2013) whereas the lowest range of Chikungunya virus dissemination was found in mosquitoes from Vietnam (Zouache et al., 2014). Indeed, the midgut microbiota is the first barrier encountered by viruses during their infection process and its diversity could strongly interferes with virus replication (Jupatanakul et al., 2014; Kenney and Brault, 2014a). However, microbiota is not the only factor that can modulate mosquito competence. Vector spatial genetic structure (gene flow, presence of cryptic species, invasion) has previously been demonstrated to greatly influence its interactions with pathogens (reviewed by McCoy, 2008; Léger et al., 2013). In *Ae. albopictus* recent studies highlighted that complex genetic-genetic-environment interactions impacted the transmission of Chikungunya virus (Zouache et al., 2014). However, the effect of mosquito genetic diversity on disease transmission remains unclear. Nevertheless, previous empirical evidences and models based on host-pathogens systems predicted that host genetic diversity can negatively affect the prevalence of pathogens (reviewed by King and Lively, 2012). It is conceivable that the reduction and change in the mosquito microbiota and its associated immune response could partly explain the efficient vector competence observed in *Ae. albopictus* populations from Metropolitan France.

In conclusion, our results suggest a similar pattern in reduction of genetic diversity of *Ae. albopictus* and bacterial microbiota diversity. This finding provides new insights into the biology of an invasive species and its associated bacterial microbiota. It also highlights the need for further ecological studies to describe how the invasive mosquito population, as well as its hologenome, responds when challenged by new biotic and abiotic factors. Moreover, the dynamics of mosquito-associated eukaryotic and viral microbiota should also be investigated to gain a fully integrated view of the holobiont pathosystem of the Asian tiger mosquito.

## Ethical issues

No ethical issues to be promulgated.

## Author contributions

This work is part of GM's PhD dissertation (supervised by PM and CV) on *Aedes albopictus* microbiota. GM, PM, and CV conceived the project and sampling design. CB, TH, GL, KL, GM, PM, CV, and VT contributed to collection of specimens. GM, CV, FT, and VT performed all molecular work and genotyping scoring. GM and CG analyzed and interpreted the genotypic data and GM analyzed and interpreted the metabarcoding data. GM wrote the article and all other authors contributed edits and comments.

## Funding

Funding for this project was provided by grants from EC2CO CNRS and CMIRA Région Rhône-Alpes. This research was also partially funded by ERA-NET BiodivERsA with the national funders ANR-13-EBID-0007-01, FWF I-1437, and DFG KL 2087/6-1 as part of the 2012-2013 BiodivERsA call for research proposals, and was carried out within the framework of GDRI “Biodiversity and Infectious Diseases in Southeast Asia.” To achieve this work, we used the computing facilities of the PRABI cluster. We also gratefully acknowledge the contribution of the DTAMB platform of the FR41 Bio-Environment and Health (University Lyon 1).

### Conflict of interest statement

The authors declare that the research was conducted in the absence of any commercial or financial relationships that could be construed as a potential conflict of interest.

## Supplementary material

Figure S1 **Prediction of the best value of *K*. According to Evanno et al. (2005), the distribution of *ΔK* (absolute values of the second-order in change of the likelihood distribution divided by the standard deviation of the likelihoods) was plotted for each value of K (number of potential sub-groups) from 2 to 7**.

Figure S2 **Index comparisons between French and Vietnamese populations according to the 3% OTU richness estimations (Chao1), α-diversity (H′) and β-diversity homogeneity (Bray-Curtis Distance to centroid)**.

Figure S3 **Prevalence of Operational Taxonomic Units (OTUs) according to their mean relative abundance in midgut samples**. Most abundant OTUs (proportion > 0.01) are named by their assignation according to naïve Bayesian classifier (Bootstrap > 80%). The prevalence and abundance of OTUs were calculated from sequences obtained from midgut samples analyzed in the study.

Figure S4 **Map of haplotypes**. The six haplotypes proportion (H_1, H_2, H_3, H_4, H_5, H_6) are represented for each site. Scale bar, 200 km. NC, Nice; PLV, Porte-lès-Valence; SP, Saint Priest; VT, Vung Tàu City; HCM, Hô Chí Minh City; BD, Bình Du'o'ng; BGM, Bù Gia Mâp.

Figure S5 **Factorial Correspondence Analysis of mosquitos' genetic structure**. Each point represents an individual from a given population. The color indicates different original population of individuals. The ordination is based on multivariate analysis of allelic frequencies of the 11 microsatellites among individuals. NC, Nice; PLV, Porte-lès-Valence; SP, Saint Priest; VT, Vung Tàu City; HCM, Hô Chí Minh City; BD, Bình Du'o'ng; BGM, Bù Gia Mâp.

Table S1 **Sample compositions for the different analyses**.

Table S2 **Microsatellite primers and information**.

Table S3 **AMOVA analysis of phylogeny based Unifrac β-diversity**.

Table S4 **Haplotypes and nucleotide diversity**.

Table S5 **Bottleneck analysis**.

Table S6 **Dominant contaminant OTUs found in the negative control**.
