# Supplementary material for: French invasive Asian tiger mosquito populations harbor reduced bacterial microbiota and genetic diversity compared to Vietnamese autochthonous relatives
Source: Front Microbiol. 2015 Sep 22;6:970. doi: 10.3389/fmicb.2015.00970 (PMC4585046; doi:10.3389/fmicb.2015.00970)
Supplement: Figure S1 — Prediction of the best value of K. According to Evanno et al. (2005), the distribution of ΔK (absolute values of the second-order in change of the likelihood distribution divided by the standard deviation of the likelihoods) was plotted for each value of K (number of potential sub-groups) from 2 to 7. [file Image1.PDF]

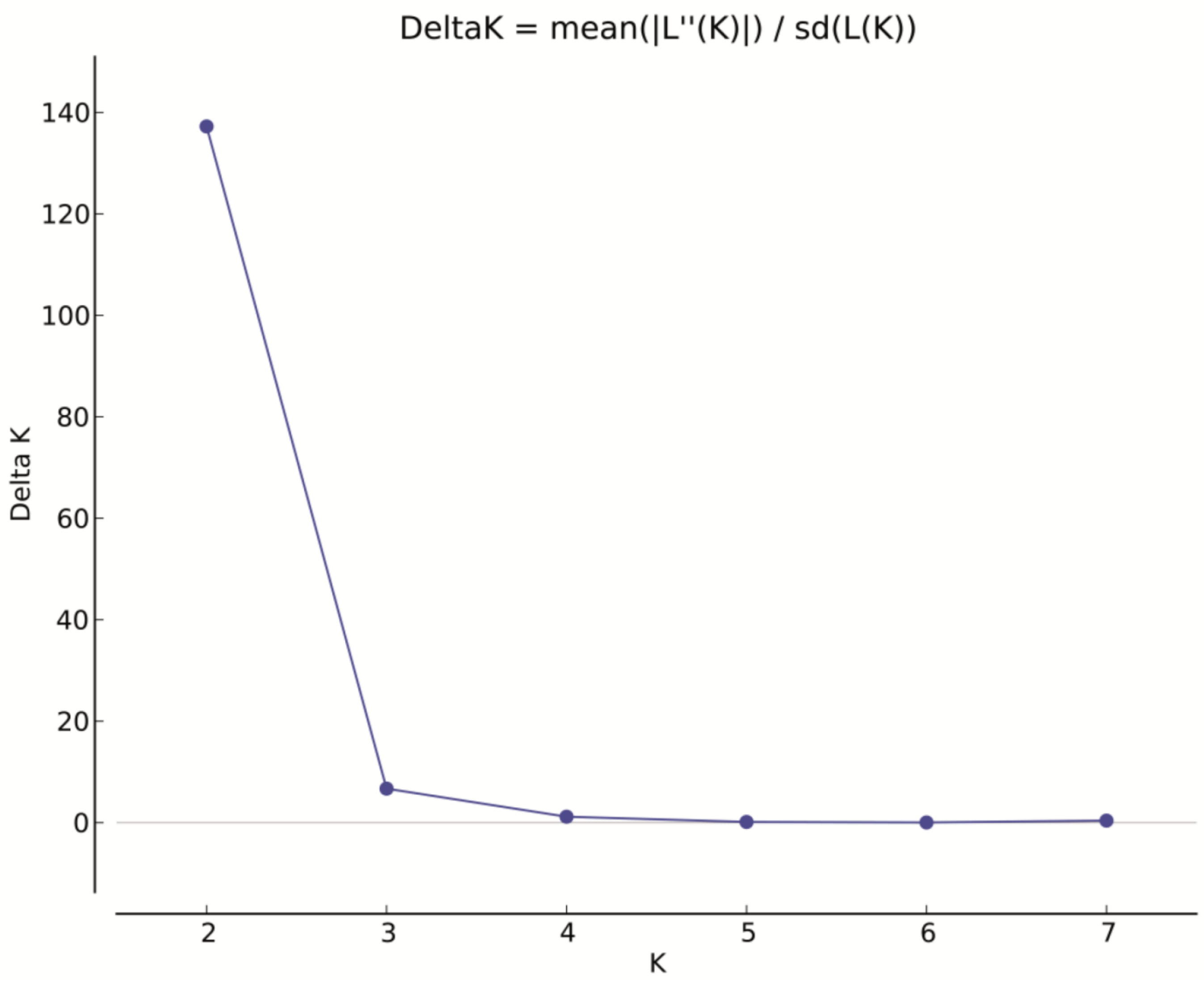

Figure S1 Prediction of the best value of *K*. according to Evanno (2005), the distribution of *ΔK* (absolute values of the second-order in change of the likelihood distribution divided by the standard deviation of the likelihoods) was plotted for each value of K (number of potential sub-groups) from 2 to 7.

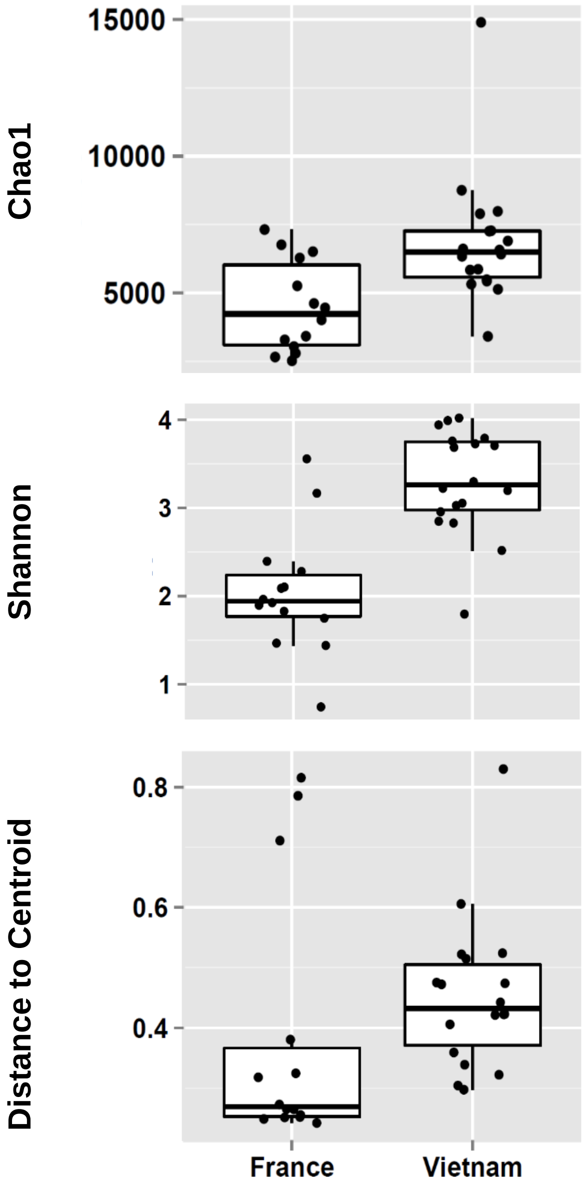

Figure S2 Index comparisons between French and Vietnamese populations according to the 3% OTU richness estimations (Chao1), α-diversity (H’) and β-diversity homogeneity (Bray-Curtis Distance to centroid).

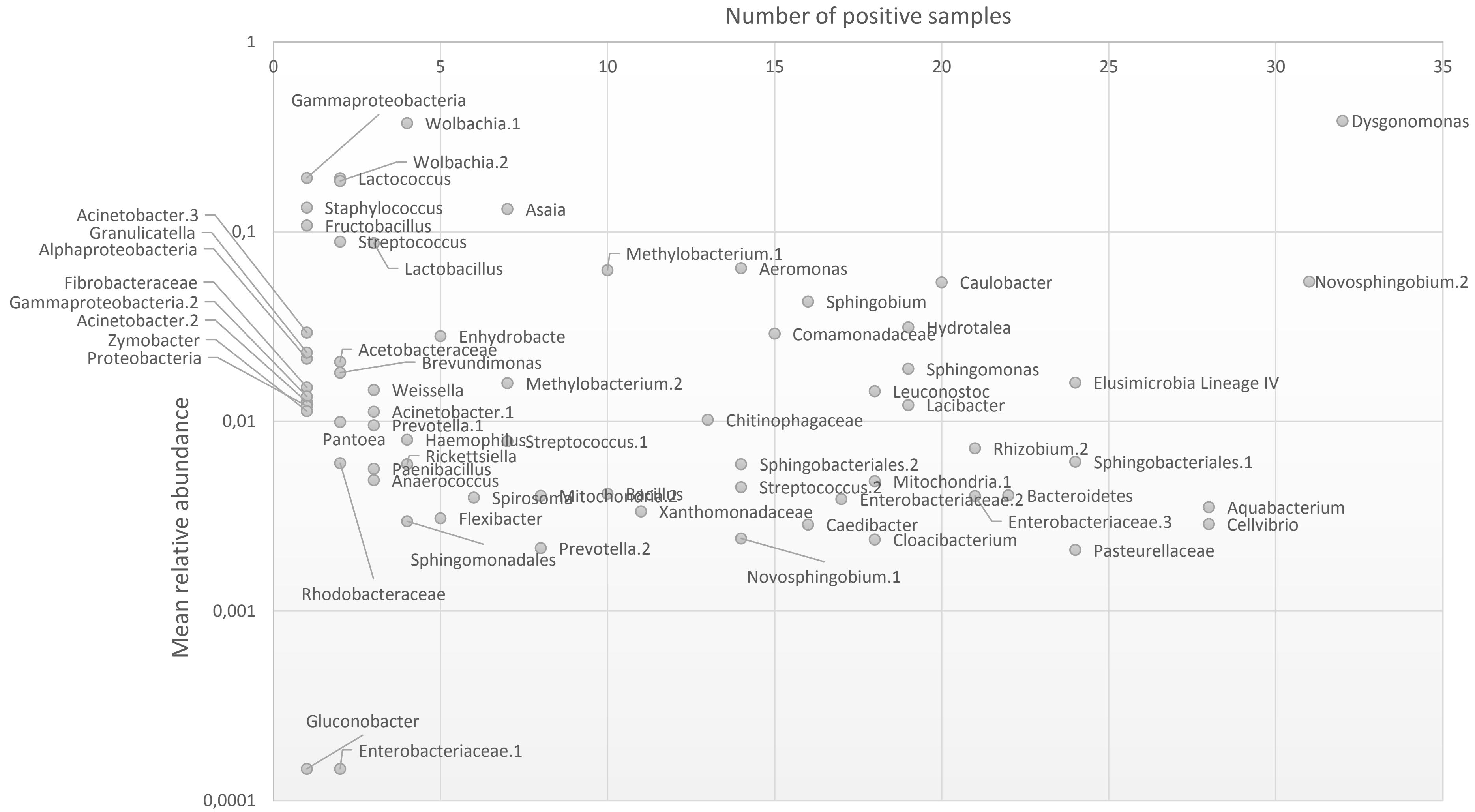

Figure S3 Prevalence of Operational Taxonomic Units (OTUs) according to their mean relative abundance in midgut samples. Most abundant OTUs (proportion > 0.01) are named by their assignation according to naïve Bayesian classifier (Bootstrap > 80%). The prevalence and abundance of OTUs were calculated from sequences obtained from midgut samples analyzed in the study.

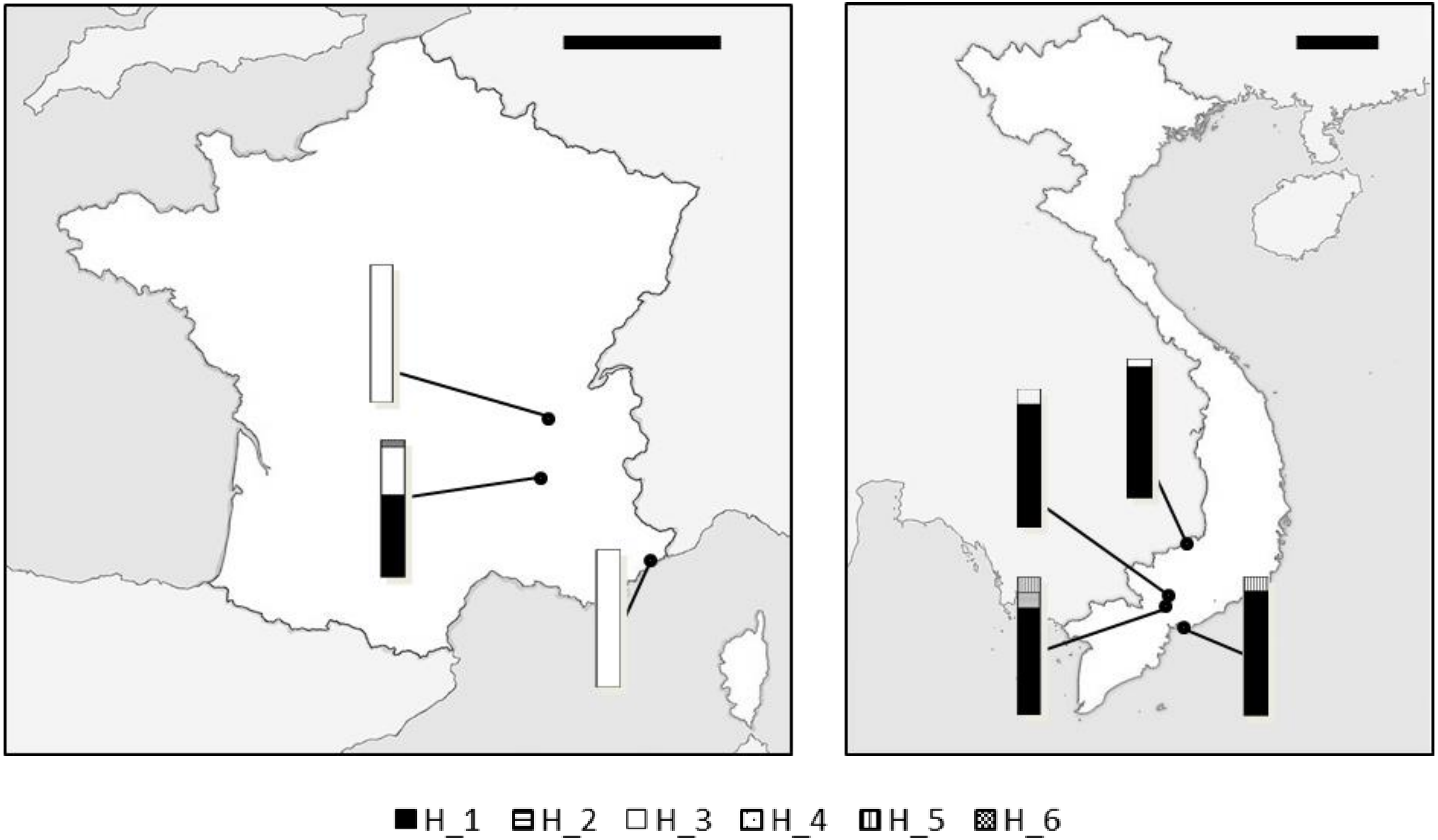

Figure S4 Map of haplotypes. The six haplotypes proportion (H_1, H_2, H_3, H_4, H_5, H_6) are represented for each site. Scale bar, 200 km. NC, Nice; PLV, Porte-lès-Valence; SP, Saint Priest; VT, Vũng Tàu City; HCM, Hồ Chí Minh City; BD, Bình Dương; BGM, Bù Gia Mập.

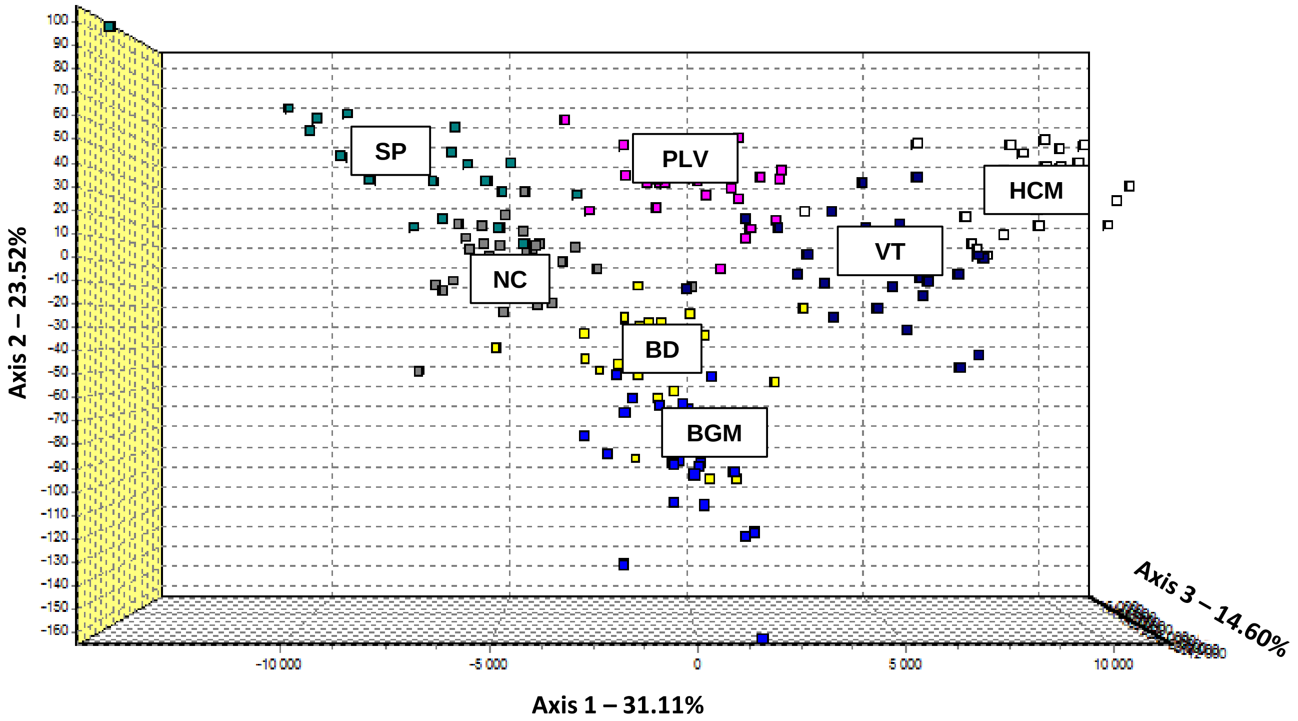

Figure S5 Factorial Correspondence Analysis of mosquitos' genetic structure. Each point represents an individual from a given population. The color indicates different original population of individuals. The ordination is based on multivariate analysis of allelic frequencies of the 11 microsatellites among individuals. NC, Nice; PLV, Porte-lès-Valence; SP, Saint Priest; VT, Vũng Tàu City; HCM, Hồ Chí Minh City; BD, Bình Dương; BGM, Bù Gia Mập.
