# Supplementary material for: French invasive Asian tiger mosquito populations harbor reduced bacterial microbiota and genetic diversity compared to Vietnamese autochthonous relatives
Source: Front Microbiol. 2015 Sep 22;6:970. doi: 10.3389/fmicb.2015.00970 (PMC4585046; doi:10.3389/fmicb.2015.00970)
Supplement: Table S1 — Sample compositions for the different analyses. [file Table1.PDF]

Table S1 Sample compositions for the different analyses.

Country	Sites	Haplotype	Microsatellites	*Wolbachia* quantification	Bacterial metabarcoding	
		Whole body	Whole body	Whole body	Individual midgut	Pooled carcasses
France	NC	10	30	10	4	1
	PLV	20	30	10	5	1
	SP	10	29	10	5	1
Vietnam	VT	10	28	10	5	1
	HCM	9	30	10	5	1
	BD	9	30	10	5	1
	BGM	17	22	10	3	1
Total		85	199	70	32	7

Table S2 Microsatellite primers and information.

Locus	Accession number	size range	5'modified-Forward	Reverse	Reference
AealbA9	DQ366022	141-179	5'PET-TGGGACAAGAGCTGAAGGAT	CTCGTTCTCTACTCTCTCCGTT	Porretta et al., 2006
AealbB51	DQ366023	132-160	5'VIC-TCCACGTGGTATAACTCTGA	GTAGTTGTCCAATTAACATCG	Porretta et al., 2006
AealbB52	DQ366024	165-176	5'NED-GGGTCTAGAAGTAATAGCGATG	GCATTCTTTGCTTCTGTTTGC	Porretta et al., 2006
AEDC	T58313	203-228	5'FAM-TGCAGGCCCAGATGCACAGCC	TCCGCTGCCGTTGGCGTGAAC	Chambers ert al., 2007
Alb-di6	KF146972	245-267	5'ATTO565-TCTTCATCTACGCTGTGCTC	GACGCCAATCCGACAAAGTC	Beebe et al., 2013
Alb-tri3	KF146973	102-134	5'Yakima Yellow-AGATGTGTCGCAATGCTTCC	GATTCGGTGATGTTGAGGCC	Beebe et al., 2013
Alb-tri18	KF146975	221-263	5'Yakima Yellow-ACACAATTGCCGTTCAGCTC	CGTCTAATAGCTCCGGTCCC	Beebe et al., 2013
Alb-tri25	KF146978	253-279	5'ATTO550-CCAACCAACAACCCAGGAAC	TACGATGCGCAACCATCATC	Beebe et al., 2013
Alb-tri41	KF146980	103-136	5'ATTO550-GATCGATTTGGGAGCTTCTG	GAACCTCTTCTCGCTTGGCT	Beebe et al., 2013
Alb-tri45	KF146982	116-143	5'ATTO565-TTTCAGCTCGGTGTTATGGC	TGATGTTGATGATGATGACTACGA	Beebe et al., 2013
Alb-tri6	KF146974	156-190	5'FAM-AGCACGAGTACAGAATGTGC	TGGCCTCCTACCGTTTATCTG	Beebe et al., 2013

Table S3 AMOVA analysis of phylogeny based Unifrac β-diversity.

	β-diversity (Unifrac weighted)			β-diversity (Unifrac unweighted)		
	df*	Variance (%)	*p*	df*	Variance (%)	*p*
Among Countries	1	17	0.02	1	8.3	$<10^{-4}$
Among Populations / Countries	5	7	0.43	5	3.3	0.42
Within populations	26	76		26	88.4	

*df, degree of freedom

Table S4 Haplotypes and nucleotide diversity.

Country	Site	n	π*	*Hd***
Vietnam	BD	9	0.00053	0.222
Vietnam	BGM	17	0.00028	0.118
Vietnam	HCM	9	0.00106	0.417
Vietnam	VT	10	0.00048	0.2
France	NC	10	0	0
France	PLV	20	0.00138	0.542
France	SP	10	0	0

* π, nucleotide diversity

** *Hd*, Haplotype diversity

Table S5 Bottleneck analysis.

Site	Wilcoxon test			Mode-Shift
	I.A.M.*	T.P.M.**	S.M.M.***	
BD	0.05	0.99	0.99	no
HCM	0.16	0.99	0.99	no
VT	0.001	0.95	0.99	no
BGM	0.07	0.97	0.99	no
NC	0.08	0.95	0.99	no
PLV	0.22	0.78	0.96	no
SP	0.005	0.79	0.99	no

*I.A.M., Infinite Allele Model

**T.P.M., Two Phase Model

***S.M.M., Stepwise Mutation Model

Table S6 Dominant contaminant OTUs found in the negative control.

OTU number	Taxonomic assignment
Otu000002	Unclassified_*Gammaproteobacteria*
Otu000004	*Chryseobacterium*
Otu000006	Unclassified_*Enterobacteriaceae*
Otu000009	*Stenotrophomonas*
Otu000010	*Acinetobacter*
Otu000011	*Pseudomonas*
Otu000019	*Flavobacterium*
Otu000025	*Chryseobacterium*
Otu000031	Unclassified_*Moraxellaceae*
Otu000036	*Pedobacter*
Otu000037	Unclassified_*Xanthomonadaceae*
Otu000038	*Chryseobacterium*
Otu000075	*Gemella*
Otu000215	*Gluconobacter*
